# Decreased HD-MIR2911 absorption in human subjects with the SIDT1 polymorphism fails to inhibit SARS-CoV-2 replication

**DOI:** 10.1038/s41421-020-00206-5

**Published:** 2020-09-11

**Authors:** Zhen Zhou, Yu Zhou, Xia-Ming Jiang, Yanbo Wang, Xi Chen, Gengfu Xiao, Chen-Yu Zhang, Yongxiang Yi, Lei-Ke Zhang, Liang Li

**Affiliations:** 1 Nanjing Drum Tower Hospital Center of Molecular Diagnostic and Therapy, State Key Laboratory of Pharmaceutical Biotechnology, Jiangsu Engineering Research Center for MicroRNA Biology and Biotechnology, NJU Advanced Institute of Life Sciences (NAILS), NJU Institute of AI Biomedicine and Biotechnology, School of Life Sciences, Nanjing University, Nanjing, Jiangsu 210023 China; 2 State Key Laboratory of Virology, Wuhan Institute of Virology, Center for Biosafety Mega-Science, Chinese Academy of Sciences, Wuhan, Hubei 430071 China; 3 Department of Critical Care Medicine and Nanjing infectious Disease Center, The Second Hospital of Nanjing, Nanjing University of Chinese Medicine, Nanjing, Jiangsu 210003 China

**Keywords:** miRNAs, Mechanisms of disease

Dear Editor,

The Coronavirus Disease 2019 (COVID-19) pandemic has been the most serious worldwide public health crisis in recent times and has been ongoing since February^1^. Most countries have relied on physical isolation or herd immunity to try to control the rapid spread of the disease^2,3^. Unfortunately, to date, neither remdesivir nor hydroxychloroquine have been shown to effectively inhibit severe acute respiratory syndrome coronavirus (SARS-CoV-2)^4,5^. While, we have found that the absorption of MIR2911 in honeysuckle decoction (HD) inhibits SARS-CoV-2 replication and accelerates the negative conversion of infected patients^6^. Therefore, HD-MIR2911 treatment will be extremely helpful in controlling the pandemic^6^. On the other hand, our mechanistic studies have demonstrated that SID1 transmembrane family member 1 (SIDT1) in the gastric pit cell membrane mediates dietary microRNA (including MIR2911 in HD) uptake into cells by using SIDT1 KO mouse model^7^. These microRNAs self-assemble into exosomes and are then secreted into the circulation and delivered into target tissues, including the liver, lung, spleen, pancreas, and T cells^7,8^. In this study, we further investigated the mechanism underlying the dysfunction of HD-MIR2911 treatment in human subjects with the SIDT1 polymorphism.

In order to assess the important rule of SIDT1 in human dietary miRNA (including MIR2911 in HD) absorption, we have first sequenced SIDT1 gene from 135 healthy volunteers. Surprisingly, 22 out of 135 volunteers have been identified a SIDT1 polymorphism (rs2271496, hereafter referred to as SIDT1poly) that leads to amino acid replacement (Val78Met) (Fig. 1a). Those results indicate that there is polymorphism at a significant frequency in human populations in the transporter gene SIDT1 (16%). To study whether this SIDT1 polymorphism can affect the uptake of exogenous miRNAs, we overexpressed SIDT1^*poly*^ and wild-type SIDT1 (SIDT1^*wt*^) in the SIDT1-deficient (SIDT1^−/−^) HEK293T cell line and then incubated the cells with 3 typical exogenous dietary miRNAs, including MIR156a, MIR168a, and MIR2911^9,10^. As shown in Fig. 1b, low pH (pH 3.5) enhanced the uptake of exogenous miRNAs in both SIDT1^*poly*^ and SIDT1^*wt*^ cells, which is consistent with the results of a previous report^7^ (Fig. 1b). SIDT1^*poly*^ cells showed a significant reduction in low pH-dependent uptake compared to SIDT1^*wt*^ cells (Fig. 1b), which is similar to that observed for SIDT1 KO cells in a previous study^7^.

**Fig. 1:**
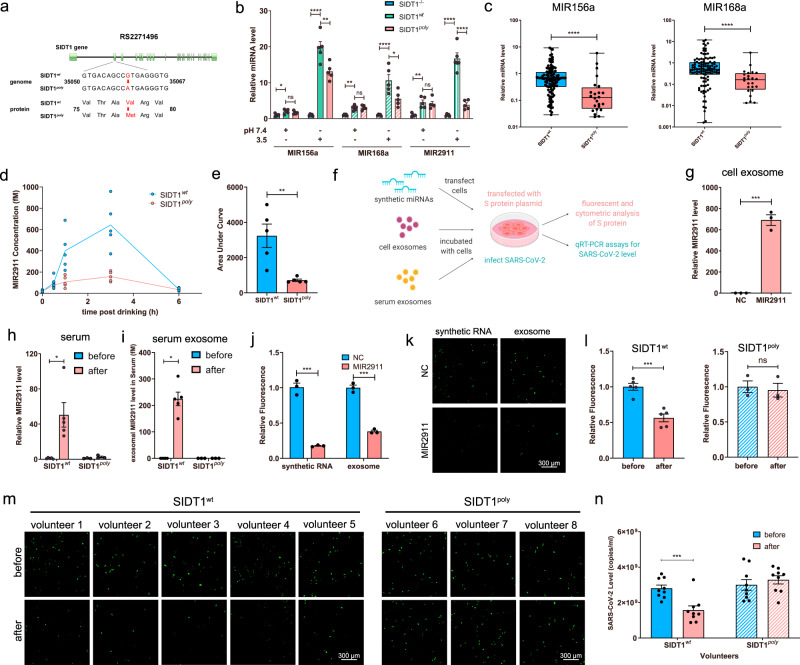
SIDT1 polymorphism decreased absorption of exogenous miRNAs. **a** Diagram of the position of SNP rs2271496 in the SIDT1 gene. **b** The miRNA levels in HEK293T cells after incubation with synthetic miRNAs at a pH of 7.4 or pH 3.5. **c** Basal level of MIR156 or MIR168 in serum samples of volunteers that carry SIDT1^*wt*^ or SIDT1^*poly*^, respectively. **d** Dynamic MIR2911 levels in serum samples from volunteers after oral administration of 200ml HD prepared from 30g dried honeysuckle. **e** Area under the curve of the dynamic MIR2911 levels. **f** Diagrams of the collection of exosomes from cell medium or serum before or after the oral administration of HD and the measurement of the biological activities of synthetic miRNAs or exosomal miRNAs in inhibiting S protein expression. **g** Relative MIR2911 level in exosomes isolated from culture mediums of HEK293T cells transfected with MIR2911 or NC miRNAs. **h**, **i** Serum MIR2911 levels (**h**) and serum exosomal MIR2911 levels (**i**) in 5 volunteers who carry SIDT1^*wt*^ and 3 volunteers who carry SIDT1^*poly*^ before and after oral administration of HD. **j**, **k** Cytometric analysis (**j**) and fluorescence images (**k**) of GFP-S-protein-overexpressing HEK293T cells after transfection with MIR2911 or NC miRNAs and incubation with exosomes collected from HEK293T cell medium after transfection with MIR2911 or NC miRNAs. **l**, Cytometric analysis of GFP-S-protein-overexpressing HEK293T cells after incubation with serum exosomes from 5 volunteers carrying SIDT1^*wt*^ (left panel) and 3 volunteers carrying SIDT1^*poly*^ (right panel) before or after oral administration of HD. **m** Fluorescence images of GFP-S-protein-overexpressing HEK293T cells after incubation with serum exosomes from 5 volunteers carrying SIDT1^*wt*^ (left panel) and 3 volunteers carrying SIDT1^*poly*^ (right panel) before or after oral administration of HD. **n** Efficacy of exosomal MIR2911 in human serum in inhibiting replication of SARS-CoV-2 before or after oral administration of HD.

To assess the impact of SIDT1^*poly*^ on RNA uptake in vivo, human blood samples were collected and divided into two groups according to the presence or absence of the SIDT1 polymorphism. Among a total of 135 volunteers, 22 volunteers were determined to be in the SIDT1^*poly*^ group, while the other volunteers were determined to be in the SIDT1^*wt*^ group. We examined the basal levels of exogenous miRNAs and showed that the serum levels of MIR156a and MIR168a in the SIDT1^*poly*^ group were 10-fold lower than those in the SIDT1^*wt*^ group (Fig. 1c). Furthermore, we analyzed the dynamic changes in serum MIR2911 in WT and polymorphic subjects after the oral administration of 200 ml honeysuckle decoction prepared from 30 g of dried honeysuckle (MIR2911 concentration: 52.5 pM). The serum MIR2911 began to increase at 1 h, reached a peak at 3 h and returned to the basal level at 6 h after consumption of HD in both WT and polymorphic subjects (Fig. 1d). Similar to the results of the in vitro study, SIDT1^*poly*^ subjects showed remarkably decreased MIR2911 absorption (AUC: approximately 5-fold) (Fig. 1d, e). Together, these data suggest that the amino acid replacement caused by SIDT1^*poly*^ compromises the capability for exogenous MIR2911 absorption in vivo, which ultimately leads to the reduction of the level of MIR2911 in blood.

Our previous study demonstrated that MIR2911 in HD, the absorption of which is mediated by SIDT1, is delivered into the lung by exosomes through the circulation^9,10^. Therefore, to confirm that the lack of response to HD treatment in SARS-CoV-2-infected patients was due to decreased MIR2911 absorption caused by the SIDT1 polymorphism, circulating exosomes from WT and polymorphic subjects before and after consumption of HD were isolated and incubated with cells expressing S-protein or SARS-CoV-2 virus (Fig. 1f). Given that we have identified 28 MIR2911 binding sites that are distributed in the SARS-CoV-2 genome and 4 sites in S-protein (Supplementary Fig. S1), the absorbed MIR2911 should directly affect S-protein expression. As shown in Fig. 1h, i, SIDT1^*poly*^ subjects showed significantly reduced absorption of MIR2911 in serum and in isolated exosomes (Fig. 1h, i). As shown in Fig. 1j, k, synthetic MIR2911 and exosomal MIR2911 collected from MIR2911-transfected cells remarkably inhibited S-protein expression, suggesting that MIR2911 indeed directly binds to S-protein mRNA and inhibits its expression (Fig. 1j, k). The exosomes containing MIR2911 (collected from WT subjects after consumption of HD) significantly inhibited S-protein expression, while MIR2911^–^ exosomes (collected from polymorphic subjects before and after consumption of HD) had no effect on S-protein expression (Fig. 1l, m). Furthermore, exosomes containing MIR2911 (collected from MIR2911-transfected cells or from WT subjects after consumption of HD) also significantly inhibited SARS-CoV-2 virus replication, but no effect of SIDT1 polymorphic exosomes was observed (Fig. 1n). The results suggest that decreased HD-MIR2911 absorption in human subjects with the SIDT1 polymorphism leads to a failure to inhibit SARS-CoV-2 replication.

Current studies support that SIDT1 mediates dietary miRNA absorption in humans. Interestingly, we conducted a clinical study in which one group of moderate (common)-type COVID-19 patients was treated with MIR2911 in HD (MIR2911^+^) in addition to routine antiviral therapy (RT)^6^. As shown in Supplementary Fig. S2, the time taken to become SARS-CoV-2 PCR-negative (TTN) for 5 patients was 3.8 days, while the TTN for one patient was 17 days (Supplementary Fig. S2). In fact, we have indeed identified same SIDT1 polymorphism in nonresponsive patients. Those data further support that SIDT1 mediates dietary miRNA absorption in human and the absorption of MIR2911 in honeysuckle decoction inhibits SARS-CoV-2 replication. In this study, we did our best to enroll more human subjects. However, we identified 51 volunteers who accepted to drink HD for genotype of SIDT1 and only 5 donors have the SIDT1 polymorphism. Studies with the larger number of human subjects would strengthen the conclusions. HD treatment will provide a practicable and reliable therapeutic strategy to treat SARS-CoV-2 infection. However, we also need to develop a new method to treat SIDT1 polymorphic patients that does not involve the consumption of HD.

## Supplementary information

Supplementary Information, Figures and Methods

## Supplementary information

**Supplementary Information** accompanies the paper at (10.1038/s41421-020-00206-5).
