# Supplementary material for: Decreased HD-MIR2911 absorption in human subjects with the SIDT1 polymorphism fails to inhibit SARS-CoV-2 replication
Source: Cell Discov. 2020 Sep 11;6:63. doi: 10.1038/s41421-020-00206-5 (PMC7484494; doi:10.1038/s41421-020-00206-5)
Supplement: Supplementary file 1 — Supplementary Information, Figures and Methods [file 41421_2020_206_MOESM1_ESM.pdf]

Supplementary Materials and Methods

Honeysuckle and honeysuckle decoction

Dry honeysuckle was bought from the local TCM store. 30 g dried herb was then boiled in 600 ml water for 1 h and 200 ml herb decoction remained at last.

Cell lines and culture conditions

HEK293T cells (ordered from Type Culture Collection of Chinese Academy of Sciences) were cultured in high glucose dulbecco's modified eagle medium (DMEM, Gibco) supplemented with 10% fetal bovine serum (FBS, Gibco) and 1% penicillin-streptomycin at 37℃ with 5% CO_2.

African green monkey kidney Vero E6 cell line was obtained from American Type Culture Collection (ATCC) and cultured in DMEM supplemented with 10% FBS at 37 °C with 5% CO_2.

Low pH treatment

The pH of the culture medium was adjusted with hydrochloric acid to pH 3.5, and an unmodified culture medium (approximately pH 7.4) was used as a control. Synthetic miRNAs were directly added to the medium with different pH values (40 pmol/ml). Then miRNA and culture medium mixture was incubated with cultured cells for 30 min. At last, the cells were washed three times and incubated with FBS-free medium containing RNase A at 37 ℃ for 1 h to remove extracellularly attached miRNAs.

Human blood Sample Collection

To detect the baseline level of exogenous miRNA in serum, we obtained 135 random adult volunteers seeking routine checkups at Jinling Hospital, Nanjing, China, according to protocols approved by the ethics committee of Jinling Hospital. All blood donors contribute one whole blood sample and one serum sample, respectively.

To test the impact of SIDT1 polymorphism on miRNA uptake, 10 volunteers were divided into two groups, the control group ($SIDT1^{wt}$, n=5) and the SIDT1 polymorphism group ($SIDT1^{poly}$, n=5). Each donor was fed 200 ml honeysuckle decoction. Serum samples were collected before and 0.5, 1, 3, 6 hours after honeysuckle decoction treatment, respectively.

To assess the function of exosomal MIR2911 in serum, 8 volunteers were divided into two groups, the control group ($SIDT1^{wt}$, n=5) and the SIDT1 polymorphism group ($SIDT1^{poly}$, n=3). Each donor was fed 200 ml honeysuckle decoction. Serum samples were collected before and 2 hours after honeysuckle decoction treatment.

Genotyping

To analyze the SNP rs2271496 of *sidt1*, genomic DNA was extracted from 100 µl whole blood sample of each volunteer and purified with the DNeasy Blood & Tissue kit (TIAGEN), according to the manufacturer’s instruction. For genotyping, we designed a pair of primers with the sequence TACAGTCCTTGGAAGAGCAGAG/TGTGCATGTGCACGTTTTGCAT. After 35 cycles of amplification by multiplex PCR, the PCR product was purified by ExoI (Fermentas) and FastAP (Fermentas) to remove excess primers and dNTPs. 2µL of purified PCR product was

mixed with 1μL Snapshot Mix (ABI), 0.1μL extension primer (CCCTGCCATGCAGGTGACAGCC) and 2.9μL milliQ water. The following extension reaction was employed: 30 cycles (96 °C for 10 sec, 52°C for 5 sec, 60°C for 30 sec). After the extension product was added to the loading buffer, it was denatured at 95 degrees Celsius for 3 minutes and ice cold immediately, then sequenced by a 3730XL sequencer (ABI).

Exosome isolation

For exosome collection from culture cells, HEK293T cells were transfected with miRNA by using Lipofectomine 2000 (invitrogen). After 36 hours, exosomes were isolated from the cell culture medium using a Total Exosome Isolation Reagent (Invitrogen) according to the manufacturer's instructions.

For human serum exosome collection, exosomes were isolated from human serum samples using a Serum Exosome Isolation Kit (Vazyme) according to the manufacturer's instructions.

Exosome pellets were collected and re-suspended in PBS.

RNA isolation and quantitative RT-PCR assays

Small RNAs (<100 nt) from honeysuckle decoction were extracted using the Universal Plant MicroRNA Kit (Biotech) according to manufacturer's instructions. Total RNAs from human serum, human exosomes, cell exosomes were extracted using TRIZOL reagent according to the manufacturer's instructions.

To detect MIR2911 level, quantitative RT-PCR was performed using TaqMan miRNA probes (Applied Biosystems) using an LC96 PCR machine according (Roche) to the manufacturer's instructions. A series of synthetic MIR2911 oligonucleotides at known concentrations was reverse transcribed and amplified to build standard curve. The absolute amount of MIR2911 was then calculated in reference to the standard curve.

miRNA target prediction

SARS-CoV-2 genome sequence was acquired from the NCBI database (Refseq ID: MN908947). RNAhybrid[1] was used to search potential MIR2911 binding sites on SARS-COV-2 S gene.

Evaluation of inhibitory effect on S protein of synthetic MIR2911 and exosomes

To test the inhibitory effect on S protein of synthetic MIR2911, HEK293 cells were transfected with GFP-S protein plasmid (Genscript) and synthetic MIR2911 or NC by using Lipofectamine 2000. At 24 h post treatment, fluorescent and cytometric analysis were employed to test the expression level of GFP-S protein.

To test the inhibitory effect on S protein of exosomes, HEK293T cells were cultured overnight in 12-well cell culture petridish and then incubated with exosomes isolated from 6 ml cell medium or 250 μl serum from different volunteers for 8 hours. The cells were then transfected with GFP-S protein plasmid by using Lipofectamine 2000. At 24 h post treatment, fluorescent and cytometric analysis were employed to test the expression level of GFP-S protein.

Evaluation of antiviral activities of the human serum exosome from volunteers

To test the antiviral efficacy of serum exosome from donors, Vero E6 cells were cultured overnight in 48-well cell-culture petridish with a density of 5×10^4 cells/well. Cells were pretreated with exosomes isolated from 2 ml cell medium or 62.5 μl serum from different volunteers for 8 hours. Subsequently, treated Vero E6 cells were infected with a clinical isolate SARS-CoV-2 (nCoV-2019BetaCoV/Wuhan/WIV04/2019[2]) at a multiplicity of infection (MOI) of 0.01. After 1 h of incubation, the virus-exosome mixture was removed and cells were washed with warm PBS and incubated in fresh medium. At 24 hours p.i., the cell supernatant was collected and lysed. The viral RNA extraction and quantitative real time PCR (RT-PCR) analysis was described in our previous study[3].

References

1 Rehmsmeier, M., Steffen, P., Hochsmann, M. & Giegerich, R. Fast and effective prediction of microRNA/target duplexes. *RNA* **10**, 1507-1517, doi:10.1261/rna.5248604 (2004).

2 Zhou, P. *et al.* A pneumonia outbreak associated with a new coronavirus of probable bat origin. *Nature* **579**, 270-273, doi:10.1038/s41586-020-2012-7 (2020).

3 Wang, M. *et al.* Remdesivir and chloroquine effectively inhibit the recently emerged novel coronavirus (2019-nCoV) in vitro. *Cell Res* **30**, 269-271, doi:10.1038/s41422-020-0282-0 (2020).

Supplementary Information, Fig. S1

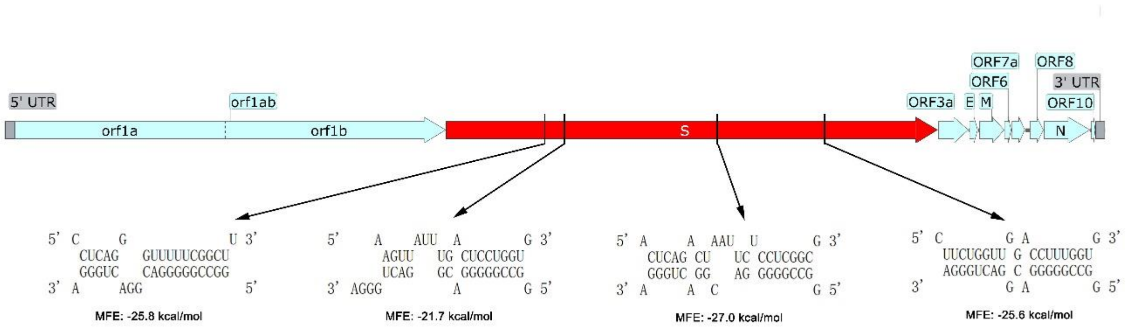

Fig. S1 Schematic description of the base pairing between MIR2911 and mRNA of SARS-CoV-2 S gene.

Supplementary Information, Fig. S2

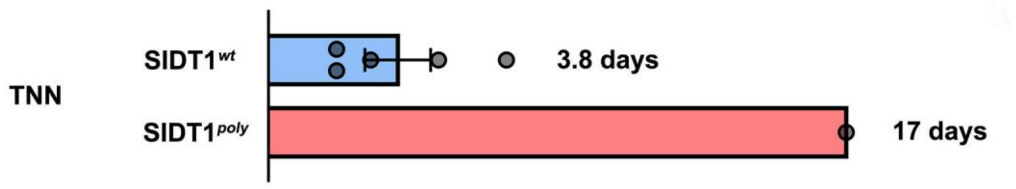

Fig. S2 The time taken to become SARS-CoV-2 negative (TTN) of patients who carry $SIDT1^{wt}$ or $SIDT1^{poly}$.
